# Green Synthesis and Characterization of Fe-Ti Mixed Nanoparticles for Enhanced Lead Removal from Aqueous Solutions

**DOI:** 10.3390/molecules30091902

**Published:** 2025-04-24

**Authors:** Shamika P. W. R. Hewage, Harshica Fernando

**Affiliations:** Department of Chemistry, Prairie View A&M University, Prairie View, TX 77446, USA; rwishvajith@pvamu.edu

**Keywords:** nanoparticles, lead, adsorption, green synthesis, dextrose, heavy metal pollution

## Abstract

Heavy metal contamination in water resources presents a significant environmental and public health challenge, with lead being particularly concerning due to its toxicity and persistence. This study reports the green synthesis of Fe-Ti mixed oxide nanoparticles (NPs) using dextrose as a green source and investigates their effectiveness in lead removal from aqueous solutions. The synthesized NPs were characterized using XRD, FTIR, XPS, SEM-EDS, and BET analysis, revealing an amorphous structure with a high surface area (292.89 m^2^ g^−1^) and mesoporous characteristics. XPS analysis confirmed the presence of mixed Fe^3+^/Fe^2+^ valence states in a Ti^4+^-rich framework, creating diverse binding sites for lead adsorption. The material exhibited optimal lead removal at pH 5, with adsorption following pseudo-second-order kinetics (R^2^ > 0.99) and a Langmuir isotherm model (R^2^ > 0.98). Maximum adsorption capacity reached 25.10 mg g^−1^ at 40 °C, showing endothermic behavior. The low point of zero charge (PZC, 0.22) and surface hydroxyl groups enabled efficient lead binding possibly through multiple mechanisms. Dose optimization studies established 6 g L^−1^ as the optimal adsorbent concentration. The synergistic combination of iron’s affinity for heavy metals and titanium’s structural stability, coupled with environmentally friendly synthesis, resulted in a promising material for sustainable water treatment applications.

## 1. Introduction

Lead (Pb) contamination in water resources represents a critical global environmental and public health concern. As a cumulative toxicant, Pb can severely impact neurological systems, kidneys, and blood circulation, with particularly devastating effects on children, infants, and fetuses [1]. The metal’s ability to distribute across vital organs, including the brain, liver, kidneys, and bones, makes it exceptionally dangerous to human health [2]. Lead’s enhanced absorption rate and interference with developmental processes pose an elevated risk in children, as they lack the bone-storage mechanism present in adults [3].

Environmental Pb contamination stems from both natural and anthropogenic sources [4]. While Pb occurs naturally in Earth’s crust, human activities have dramatically increased its environmental presence through mining operations, industrial processes, leaching from landfills, and agricultural practices [5]. In water systems, Pb enters through various pathways, including industrial effluents, corrosion of Pb-containing infrastructure, and surface runoff from contaminated soils [6]. Recognizing these risks, the U.S. Environmental Protection Agency recently lowered the Pb action level in drinking water from 15 to 10 parts per billion (ppb) through the Pb and Copper (Cu) Rule Improvements in October 2024 [7]. Multiple techniques have been developed for Pb removal from water, including chemical precipitation, ion exchange, membrane filtration, and adsorption [8]. Among these, adsorption has emerged as a particularly promising approach due to its cost effectiveness, operational simplicity, and high efficiency [9,10]. Various adsorbents have been investigated, with metal oxides like manganese oxide (MnO_2_), iron oxide (Fe_2_O_3_), titanium dioxide (TiO_2_), and zinc oxide (ZnO) showing superior performance compared to traditional materials like activated carbon due to their enhanced binding capabilities with Pb ions [11,12].

Metal oxide nanoparticles (NPs) have garnered significant attention due to their large surface area, high reactivity, and superior adsorption capabilities [13]. Many studies have been carried out using activated carbon [14]. In addition, several synthesis methods have used green sources to make NPs to remove lead [15]. Iron oxide NPs and titanium oxide NPs have been widely used in many studies related to biomedical applications, including magnetic resonance imaging (MRI) contrast agents, drug delivery systems, tissue engineering, and environmental remediation [16]. Combining Fe oxide NPs and TiO_2_ NPs, compared to individual metal oxide NPs lies in the potential for enhanced properties and functionalities due to synergistic interactions between the different metal components, often leading to improved catalytic activity, better stability, or tailored optical properties depending on the application [17,18,19]. Mixed metal NPs have been used in previous studies, and Fe-Ti mixed particles were made using precursor solutions of Fe (II) chloride tetrahydrate and Ti oxyacetylacetonate, which were then synthesized via an aerosol-assisted chemical vapor deposition technique which have been used in the simultaneous removal of arsenic, cadmium, Cu, Pb, and fluorine ions from water solutions [20]. Combining Fe and Ti oxides is auspicious as it synergistically combines Fe’s high affinity for heavy metals with Ti’s structural stability and chemical resistance [21]. However, conventional synthesis methods for these NPs often involve harsh chemicals and energy-intensive conditions, raising environmental concerns. Green synthesis approaches, utilizing environmentally benign reducing agents like natural sugars, offer a sustainable alternative while potentially enhancing surface properties for improved adsorption [22,23]. Simple sugars like glucose and sucrose have demonstrated remarkable capabilities in the controlled synthesis of various metal NP’s, offering environmentally benign alternatives to harsh chemical reducing agents [24,25,26]. For instance, white sugar has been successfully employed in silver NP synthesis under mild conditions [24]. At the same time, glucose has proven effective in the controlled formation of gold NP’s with well-defined crystalline structures [25]. In Cu NP synthesis, dextrose’s reducing capability has been utilized through a stepwise reduction mechanism, demonstrating the versatility of sugar-based green synthesis approaches [26]. The use of dextrose as a reducing agent not only promotes NP formation under mild conditions but also introduces beneficial surface functionalization that can enhance metal binding capacity. While plant extracts have emerged as environmentally friendly reducing and capping agents in NP synthesis, their application often results in lower yields compared to conventional chemical methods due to their milder reducing capabilities [27]. This limitation and the availability of plant extracts on a large scale presents a significant challenge for large-scale production and commercial viability. In contrast, simple sugars like dextrose offer an optimal balance between green synthesis principles and production efficiency. Dextrose, an inexpensive and readily available reducing agent, can facilitate higher NP yields under mild conditions while maintaining the benefits of environmentally benign synthesis. The combination of cost effectiveness, improved yield, and sustainable synthesis makes dextrose-based NP production particularly attractive for potential commercial applications in water treatment systems. Compared to traditional synthesis typically conducted at 180–220 °C, our dextrose-mediated process operates at just 60 °C, representing a 67–73% reduction in energy requirements. The elimination of harsh reduction not only reduces synthesis costs by approximately 40–50% but also dramatically decreases environmental risks, as these conventional reagents are associated with high toxicity [20].

Despite the promising attributes of metal oxide nanoparticles for heavy metal removal, significant research gaps remain in developing environmentally sustainable synthesis routes for mixed metal oxide systems with enhanced lead adsorption capabilities. While single metal oxides like iron oxide [28] and titanium dioxide [29] have been extensively studied, the synergistic potential of Fe-Ti mixed oxide NP’s remains underexplored. Conventional synthesis methods often involve harsh chemicals and energy-intensive conditions [30], creating environmental concerns that contradict the remediation goals. Furthermore, the underlying mechanisms governing lead adsorption onto mixed metal oxide surfaces, particularly the role of mixed valence states and surface functional groups, are not fully understood [13]. Although biochar-based composites have shown promise for lead removal [10], greener approaches to synthesizing mixed metal oxide NP’s with high surface area and abundant active sites for direct application in water treatment systems remain limited. This study addresses these gaps by developing a green synthesis route for Fe-Ti mixed oxide NP’s using dextrose as a reducing agent and investigating their lead removal performance under various environmental conditions.

In this study, we report the synthesis of Fe-Ti mixed oxide NPs using a green approach and investigate their effectiveness in lead removal from aqueous solutions. The work focuses on understanding the influence of various operational parameters, including pH, contact time, temperature, and adsorbent dosage, while elucidating the mechanisms of lead adsorption through comprehensive material characterization and adsorption studies.

## 2. Results and Discussion

### 2.1. Characterization of Iron/Titanium Oxide Nanoparticles

#### 2.1.1. Scanning Electron Microscopy (SEM) and Energy Dispersive X-Ray Spectroscopy (EDS) Measurements

**Figure 1:**
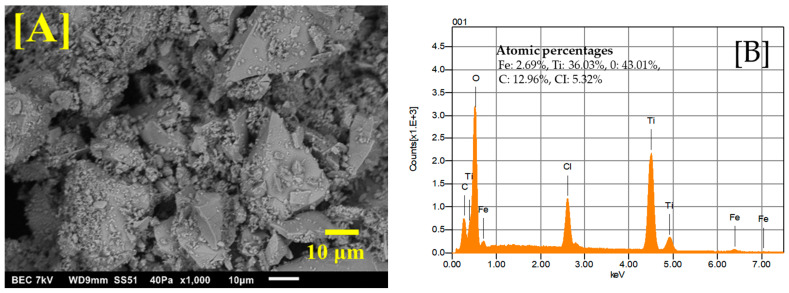
SEM image of Fe-Ti mixed NPs under ×1000 magnification (**A**) and EDS energy profile of Fe-Ti mixed NPs under ×1000 magnification (**B**).

The Fe-Ti mixed oxide NPs’ morphological characteristics and elemental composition were investigated using SEM and EDS. Figure 1A shows the SEM image obtained at ×1000 magnification, revealing a heterogeneous surface morphology with irregular aggregates. The particles exhibit a rough surface texture with varied particle sizes, forming interconnected structures with visible void spaces between aggregates. These interstitial spaces and surface roughness could enhance the material’s adsorption capacity by providing additional binding sites and increasing the accessible surface area for metal ion interactions.

EDS analysis (Figure 1B) confirmed the successful synthesis of Fe-Ti mixed oxide NPs with atomic percentages of Fe (2.69%), Ti (36.03%), and oxygen (O) (43.01%). The presence of carbon (12.96%) can be attributed to the organic residues from the dextrose used in the green synthesis process, while chlorine (5.32%) likely originates from the FeCl_3_ precursor. The atomic ratio of Ti: Fe was ~13:1. Similar heterogeneous surface morphology with irregular aggregates has been reported by [31] for Fe-modified TiO_2_ prepared by the sol–gel method, where grain size and shape were found to depend significantly on synthetic conditions and iron precursors used. Our study’s interconnected structures with void spaces align with observations by Lagos et al. [32], who reported varied morphologies in Fe-Ti oxide nanostructures with different Fe/Ti ratios. Additionally, surface roughness in our samples resembles features reported by researchers studying Fe-doped TiO_2_ NPs, where EDS analysis confirmed the effective incorporation of Fe into the TiO_2_ framework, leading to enhanced functional properties [32,33].

#### 2.1.2. Fourier Transform Infrared Spectroscopy (FTIR) Analysis

**Figure 2:**
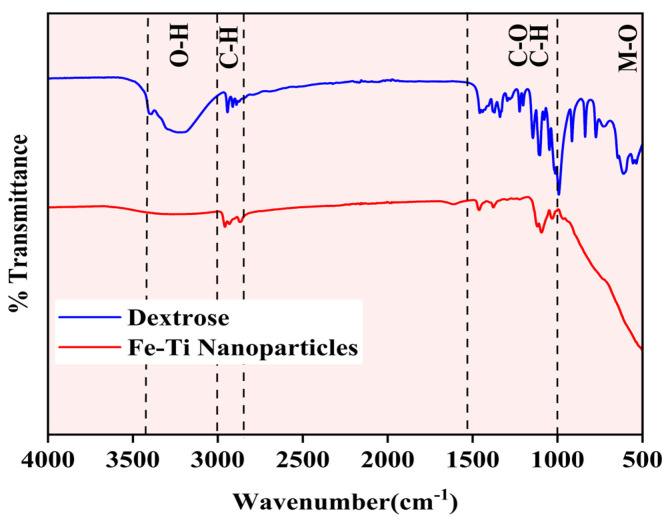
FTIR spectra of Fe-Ti mixed oxide NPs (red) and dextrose precursor (blue).

The synthesized Fe-Ti mixed oxide NP surface chemical composition and functional groups were characterized using FTIR spectroscopy and compared with the dextrose precursor spectrum (Figure 2). The spectra reveal distinct structural transformations during the synthesis process. The dextrose spectrum (blue line) exhibits characteristic absorption bands of carbohydrate functional groups: a broad, intense band at approximately 3300 cm^−1^ (O-H stretching), C-O stretching vibrations around 1500–1000 cm^−1^, C-H bending modes near 1000 cm^−1^, and multiple sharp peaks in the fingerprint region (1000–500 cm^−1^) corresponding to the dextrose ring structure [34]. The Fe-Ti mixed oxide NP spectrum (red line) shows significant modifications in peak intensity and position, indicating the successful transformation of the precursor material. The reduced intensity of the O-H stretching band (3300 cm^−1^) suggests partial dehydration during the synthesis process while retaining sufficient surface hydroxyl groups for metal binding. The attenuation of C-O (1200 cm^−1^) and C-H bending (1000 cm^−1^) vibrations indicates effective decomposition of the organic framework. Most notably, the appearance of new broad absorption bands in the low-frequency region (below 1000 cm^−1^) can be attributed to metal–oxygen (M-O) stretching vibrations, precisely forming Fe-O and Ti-O bonds in the NP structure [35]. The spectral comparison confirms the successful conversion of organic precursor to metal oxide NPs while maintaining surface hydroxyl groups that can serve as active sites for metal Fe adsorption.

#### 2.1.3. X-Ray Photoelectron Spectroscopy (XPS)

**Figure 3:**
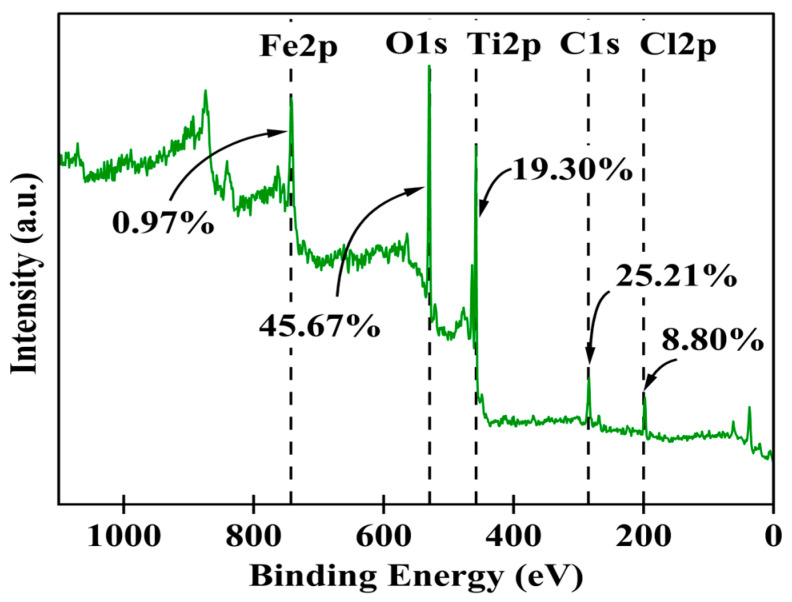
XPS survey scans for Fe-Ti mixed oxide NPs.

The synthesized Fe-Ti mixed NP surface chemical composition and electronic states were comprehensively investigated using XPS. The survey spectrum (Figure 3) revealed the presence of Fe, Ti, O, C, and Cl as the main elements on the surface, with relative atomic percentages of 0.97%, 19.30%, 45.67%, 25.21%, and 8.80%, respectively. The high oxygen content (45.67%) coupled with both Fe and Ti confirms the successful formation of mixed metal oxides. In comparison, the C content (25.21%) suggests surface functionalization or residual organic species from the green synthesis process. The presence of Cl (8.80%) can be attributed to residual precursor species from the FeCl_3_ used in synthesis.

**Figure 4:**
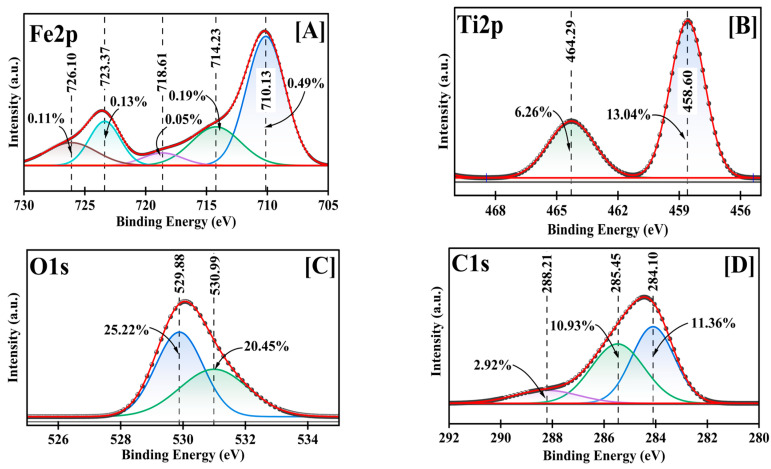
Fe2p (**A**), Ti2p (**B**), O1s (**C**), and C1s (**D**) deconvoluted high-resolution XPS for Fe-Ti mixed oxide NPs.

A detailed chemical state analysis was conducted using high-resolution XPS (Figure 4, supplementary Table S1). The C1s spectrum was initially used for charge referencing, with the adventitious carbon (C-C) peak observed at 284.10 eV. This peak was calibrated to the standard reference value of 284.40 eV for C-C bonds [36], establishing a positive shift of 0.30 eV that was subsequently applied to all measured binding energies. After calibration, the C1s spectrum revealed three distinct carbon environments: C-C bonds at 284.40 eV (11.36%), C-O bonds at 285.75 eV (10.93%), and carboxyl groups (O-C=O) at 288.51 eV (2.92%), consistent with previously reported values for carbon functionalities in metal oxide NPs [36,37].

The Fe2p spectrum revealed multiple chemical environments characteristic of mixed-valence Fe-oxide states. After calibration, the primary Fe2p3/2 peak at 710.13 eV (0.49%) shifted to 710.43 eV, corresponding to Fe^3+^ in an oxide environment [28]. This was accompanied by satellite peaks at 718.61 eV (0.19%) and 723.37 eV (0.13%), which shifted to 718.91 eV and 723.67 eV, respectively, after calibration. Interestingly, despite using FeCl_3_ as the Fe precursor, the presence of Fe^2+^ was evidenced by the peak at 726.37 eV (0.11% after calibration), indicating the partial reduction of Fe^3+^ during synthesis. This reduction can be attributed to the reducing environment created by dextrose during the green synthesis process, where the dextrose can facilitate the conversion of Fe^3+^ to Fe^2+^ [38].

The presence of mixed-valence iron species (Fe^3+^/Fe^2+^) in the final product suggests the formation of a complex Fe oxide structure favorable for adsorption applications [37]. The low overall Fe content (0.97%) indicates that Fe species maintain diverse oxidation states crucial for adsorption properties, though present in smaller quantities. The Ti2p region exhibited a well-defined doublet structure characteristic of Ti^4+^ species, with Ti2p3/2 at 458.60 eV (13.04%) and Ti2p1/2 at 464.29 eV (6.26%), which shifted to 458.90 eV and 464.59 eV, respectively, after calibration. These binding energies align with previously reported values for TiO_2_ in anatase form [29]. The significant Ti content (19.30%) and its single oxidation state indicate that Ti^4+^ forms the primary framework of the mixed oxide structure.

The O1s spectrum was deconvoluted into two distinct oxygen environments: metal-oxide bonds (M-O) at 529.88 eV (25.22%) shifted to 530.18 eV, attributed to lattice oxygen, and surface hydroxyl groups at 530.99 eV (20.45%) shifted to 531.29 eV, corresponding to adsorbed oxygen species [39]. The high total oxygen content (45.67%) indicates extensive surface oxidation and hydroxylation, beneficial for metal ion adsorption.

The comprehensive XPS analysis reveals a complex surface structure where Ti^4+^ species form the primary oxide framework, complemented by Fe^3+^/Fe^2+^ centers that create diverse binding sites. The surface chemistry is characterized by multiple adsorption mechanisms: (1) electrostatic interactions with charged surface sites, (2) surface complexation with hydroxyl groups, (3) ion exchange at defect sites, and (4) interactions with C-containing functional groups. Despite the relatively low iron content, multiple iron oxidation states suggest that iron species play a crucial role in creating active sites for metal ion adsorption. These surface characteristics support the material’s observed high efficiency in Pb^2+^ removal through multiple binding mechanisms.

The relatively high Ti: Fe atomic ratio (~13.4:1) aligns with the EDS findings, confirming that Ti forms the primary framework of the mixed oxide structure. The O content (43.01%) is consistent with the formation of metal oxides, supporting the successful synthesis of the mixed oxide system. The presence of these elements in the specified proportions suggests forming a Ti-rich mixed oxide framework with incorporated Fe species, which is favorable for creating diverse adsorption sites for heavy metal removal.

#### 2.1.4. X-Ray Diffraction Measurements (XRD) and Thermogravimetric Analysis (TGA)

**Figure 5:**
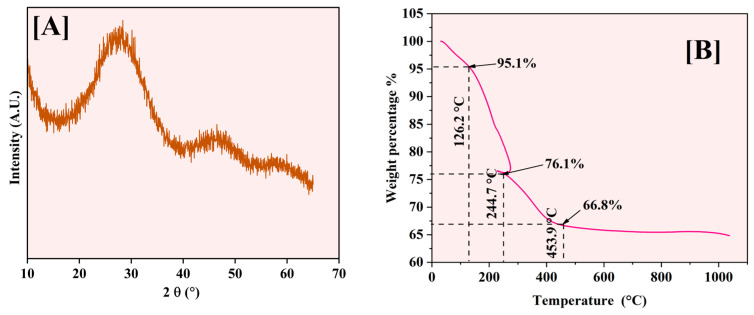
XRD pattern (**A**) and TGA analysis (**B**) of Fe-Ti mixed NPs.

XRD analysis was performed to investigate the crystalline structure of the synthesized Fe-Ti mixed oxide NPs. Figure 5A displays the XRD pattern obtained for the samples, revealing a characteristic broad diffraction peak centered at 2θ = 25–30°. This peak’s vast and diffuse nature, coupled with the absence of sharp crystalline reflections, indicates that the synthesized material predominantly exists in an amorphous state with limited long-range structural order. This low crystallinity can be attributed to the mild synthesis conditions employed in the green synthesis method, where the drying temperature (60 °C) was insufficient to promote extensive crystallization. Additionally, incorporating Fe species into the Ti-oxide framework likely introduces structural disorder, further contributing to the amorphous character of the material [30].

The observed amorphous nature of the Fe-Ti mixed oxide NPs aligns with the green synthesis approach utilized, where dextrose as a reducing agent and stabilizing molecule may interfere with crystal growth and organization during the formation process. The lack of distinct crystalline phases suggests potential advantages for adsorption applications, as amorphous materials typically exhibit higher surface areas and more accessible binding sites than their crystalline counterparts, potentially enhancing their effectiveness in heavy metal removal processes [40]. The XRD data suggest the size of the NPs around 1 (1.03) nm.

The thermal behavior of Fe-Ti mixed oxide NPs was analyzed (Figure 5B) using TGA from room temperature to 1000 °C, with distinct weight loss stages occurring at specific temperatures. The initial weight loss of 4.9% occurs at 126.2 °C, corresponding to removing physically adsorbed and surface-bound water molecules. A significant weight loss of 19% is observed at 244.7 °C, characterized by a sharp transition and endothermic loop, representing the decomposition of organic matter from dextrose and a phase transition related to the crystallization of amorphous phases.

Around 453.9 °C, a further weight loss of 9.3% is observed, bringing the total mass to 66.8% of the initial weight. This stage corresponds to the completion of organic matter decomposition and the formation of the crystalline metal oxide framework. Beyond 500 °C, the material shows excellent thermal stability with minimal weight change (≈1%), indicating the formation of stable Fe-Ti mixed oxide phases.

The endothermic loop around 244.7 °C is particularly significant, representing a phase transition where the material temporarily absorbs heat energy while maintaining relatively constant mass. This thermal event coincides with the decomposition of organic matter from the dextrose and the initiation of metal oxide framework crystallization, suggesting significant structural reorganization that influences the material’s crystallinity and surface properties [41].

#### 2.1.5. Brunauer–Emmett–Teller (BET) Surface Area Analysis

**Figure 6:**
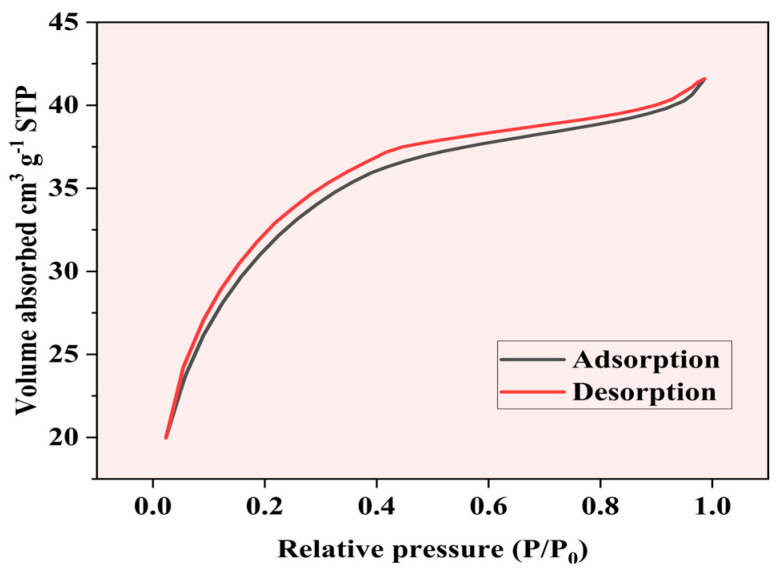
BET nitrogen adsorption–desorption.

The nitrogen adsorption–desorption isotherm of Fe-Ti mixed oxide NPs was measured at 77 K to determine the surface area and porosity characteristics. The isotherm (Figure 6) exhibits a Type IV pattern according to the IUPAC classification, which is characteristic of mesoporous materials [42]. The adsorption branch shows a gradual increase in nitrogen uptake at low relative pressures (P/P_0_ < 0.2), indicating the initial formation of a monolayer. A steeper increase in adsorption is observed in the relative pressure range of 0.2–0.4, suggesting the onset of multilayer formation and capillary condensation in mesopores [43].

The BET surface area was calculated to be 292.89 m^2^ g^−1^, with a correlation coefficient (R) of 0.99858, indicating excellent linearity in the BET plot. The C constant value of 242.885 suggests strong adsorbate–adsorbent interactions. The material exhibits an average particle diameter of 9.3116 nm and an average pore diameter of 2.3802 nm, confirming its mesoporous nature. The relatively small pore size coupled with the substantial surface area provides an optimal combination for effective adsorption processes.

The hysteresis loop observed between the adsorption and desorption branches (P/P_0_ = 0.4–1.0) is characteristic of H3-type hysteresis, typically associated with slit-shaped pores or plate-like particles creating aggregated structures [44]. The near-parallel nature of the adsorption and desorption branches suggests uniform pore size distribution within the mesoporous range. The adsorption data show a consistent increase in volume adsorbed with relative pressure, ranging from 61.88 cm^3^/g at P/P_0_ = 0.0501 to 91.90 cm^3^/g at P/P_0_ = 0.2920, demonstrating systematic pore-filling behavior.

#### 2.1.6. Evaluation of the Surface Charge of the NPs

**Figure 7:**
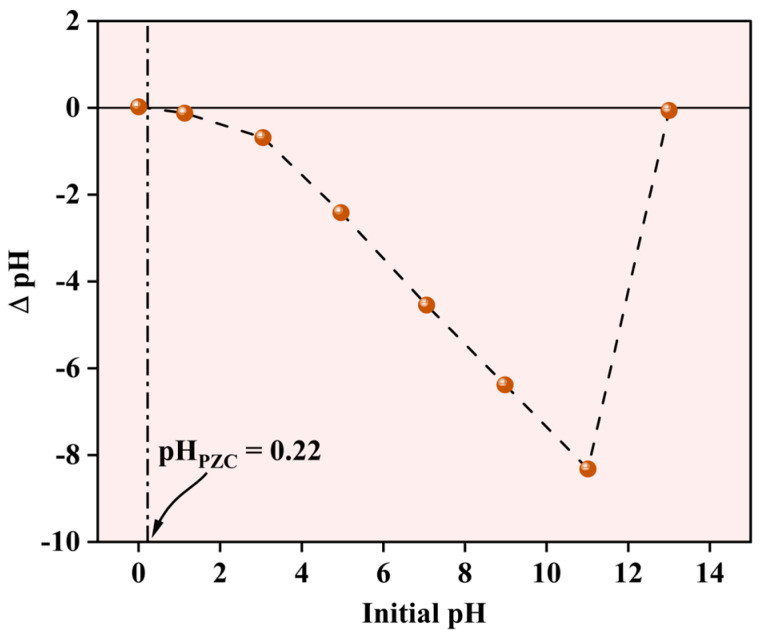
Point of zero charge for Fe-Ti mixed oxide NPs.

The pH-dependent surface characteristics of the Fe-Ti mixed oxide NPs were evaluated using the pH drift method to determine the point of zero charge (PZC). As shown in Figure 7, the PZC was determined to be 0.22, indicating the pH at which the net surface charge of the material becomes zero. The ΔpH versus initial pH plot demonstrates characteristic surface charging behavior across the studied pH range (0–14). At pH values below the PZC (0.22), the surface exhibits a slight positive charge due to the protonation of surface (X) hydroxyl groups (X-OH_2_^+^). However, at pH values above 0.22, which encompasses the most environmentally relevant conditions, the surface maintains a net negative charge due to the deprotonation of surface hydroxyl groups (X-O^−^). The pH drift curve exhibits distinct behavior in different pH regions. Between pH 2–12, a linear decrease in ΔpH is observed, suggesting consistent surface charging characteristics. The curve shows a sharp transition at highly alkaline conditions (pH 12–14), possibly indicating surface restructuring or changes in the protonation–deprotonation equilibria of surface functional groups. The notably low PZC value (0.22) implies that the synthesized NPs possess a negative surface charge under typical water treatment conditions (pH 5–8), making them particularly suitable for adsorption of positively charged metal ions such as Pb^2+^. This surface charging behavior aligns with surface hydroxyl groups identified in the XPS analysis and plays a crucial role in the electrostatic interactions governing the metal ion adsorption process.

### 2.2. Pb Studies

#### 2.2.1. Effect of Initial pH on Pb^2+^ Adsorption on Fe-Ti Mix NPs

**Figure 8:**
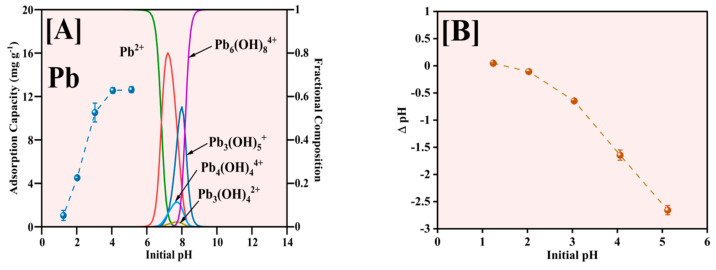
Effect of initial pH for Pb^2+^ adsorption (**A**) and pH changes after the adsorption (**B**).

The influence of solution pH on Pb^2+^ adsorption by the Fe-Ti mixed oxide NPs was comprehensively investigated in the pH range of 1–5, with results illustrated in Figure 8. The pH range was explicitly limited to pH 5 to avoid the complications arising from Pb hydroxide species formation and precipitation at higher pH values, as indicated by Pb speciation data [45]. The adsorption capacity demonstrates a strong pH dependence (Figure 8A), increasing markedly from approximately 1 mg g^−1^ at pH 1 to 12.5 mg g^−1^ at pH 5. This enhancement in adsorption capacity correlates directly with the surface charging characteristics of the Fe-Ti mixed oxide NPs. Given the material’s PZC of 0.22, the NP surface maintains a negative charge across the studied pH range, with the negative charge density increasing at higher pH values. This progressive increase in negative surface charge enhances electrostatic attraction with Pb^2+^ ions, improving adsorption performance.

The Pb speciation analysis reveals that Pb^2+^ is the predominant species below pH 6 [46], making our chosen pH range ideal for adsorption studies. At pH values above 6, the formation of various Pb hydroxide (OH) species, including Pb(OH)^+^, Pb(OH)_2_, Pb(OH)_3_^−^, and Pb(OH)_4_^2−^, becomes significant, which would complicate the interpretation of adsorption mechanisms due to potential precipitation effects [47]. The optimal adsorption performance observed at pH 5 can be attributed to several factors: the predominantly negative surface charge of the Fe-Ti mixed oxide NPs (pH > PZC of 0.22), the presence of abundant surface OH groups identified by XPS (O1s peak at 531.29 eV), and the exclusive presence of Pb^2+^ species without OH interference. Additionally, the synergistic effect of Fe^3+^/Fe^2+^ surface sites, as evidenced by XPS analysis, creates diverse binding environments that enhance the adsorption capacity.

The pH changes after adsorption (Figure 8B) reveal consistently negative ΔpH values, with the magnitude increasing from pH 1 to 5. This observation suggests that the adsorption mechanism predominantly involves surface complexation between Pb^2+^ and the deprotonated surface OH groups (M-O^−^), supported by ion exchange processes at Fe^3+^/Fe^2+^ active sites. The release of H^+^ ions during metal binding is consistent with the surface OH groups identified in the XPS analysis, indicating that the adsorption process involves electrostatic attraction and surface complexation mechanisms. Therefore, pH 5 represents the optimal condition balancing favorable surface charge characteristics, Pb speciation, and accessibility of binding sites on the Fe-Ti mixed oxide NP surface.

#### 2.2.2. Effect of Contact Time on Pb^2+^ Adsorption

**Figure 9:**
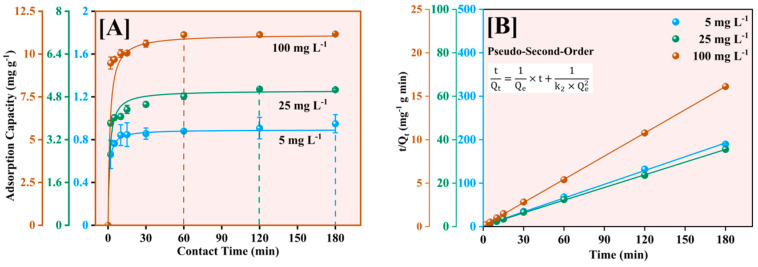
Adsorption kinetics of Pb^2+^ on Fe-Ti mixed nanoparticles at 5, 25, and 100 mg L^−1^ initial Pb^2+^ concentrations using the pseudo-second-order nonlinear (**A**) and linear (**B**) models.

The kinetics of Pb^2+^ adsorption onto Fe-Ti mixed oxide NPs was investigated using three initial concentrations (5, 25, and 100 mg L^−1^). The pseudo-second-order kinetic model was selected to consistently provide the most accurate representation of NP adsorption processes, particularly for metal ion removal applications [48]. The model was applied in both its nonlinear form (dq/dt = k_2_(Q_e_ − q)^2^) and linear form (t/q = 1/(k_2_Q_e_^2^) + t/Q_e_) to understand the adsorption kinetics [49,50] comprehensively (Figure 9).

As shown in Figure 9A, the adsorption process exhibited rapid initial uptake within the first 30 min, followed by a gradual approach to equilibrium. The equilibrium adsorption capacities reached 0.89, 5.03, and 11.15 mg g^−1^ for initial concentrations of 5, 25, and 100 mg L^−1^, respectively. The nonlinear model showed excellent fit with R^2^ values of 0.99994, 0.99759, and 0.9996 for 5, 25, and 100 mg L^−1^, respectively. The linear transformation of the model (Figure 9B) yielded even better fits with correlation coefficients (R^2^) of 0.99994, 0.9991, and 0.99963, supporting the model’s robustness in describing the adsorption process.

The calculated equilibrium adsorption capacities (Q_e_) of 0.89251 ± 0.00364, 5.03545 ± 0.10580, and 11.14685 ± 0.15850 mg g^−1^ showed excellent agreement with experimental values. The pseudo-second-order rate constants (k_2_) exhibited a clear concentration dependence: 1.25716 ± 0.07831 g mg^−1^ min^−1^ (5 mg L^−1^), 0.14987 ± 0.04240 g mg^−1^ min^−1^ (25 mg L^−1^), and 0.05968 ± 0.01159 g mg^−1^ min^−1^ (100 mg L^−1^). This systematic decrease in k_2_ with increasing concentration indicates that the initial binding site availability significantly influences adsorption kinetics, a phenomenon commonly observed in NP-based adsorption systems [51]. The high correlation coefficients and good agreement between predicted and experimental values indicate that chemisorption is the predominant rate-controlling mechanism, involving valence forces through sharing or exchanging electrons between the adsorbent and adsorbate [50]. The rate constants (k_2_) exhibited a decreasing trend with increasing initial concentration, suggesting that at lower concentrations, the abundance of available binding sites relative to Pb^2+^ ions leads to faster adsorption kinetics. The varied model fitting quality across concentrations suggests that while chemical interactions dominate at low concentrations, the adsorption mechanism becomes more complex at higher concentrations, potentially involving multiple simultaneous processes such as surface diffusion and mass transfer limitations.

#### 2.2.3. Isotherm Study on Pb^2+^ Adsorption

**Figure 10:**
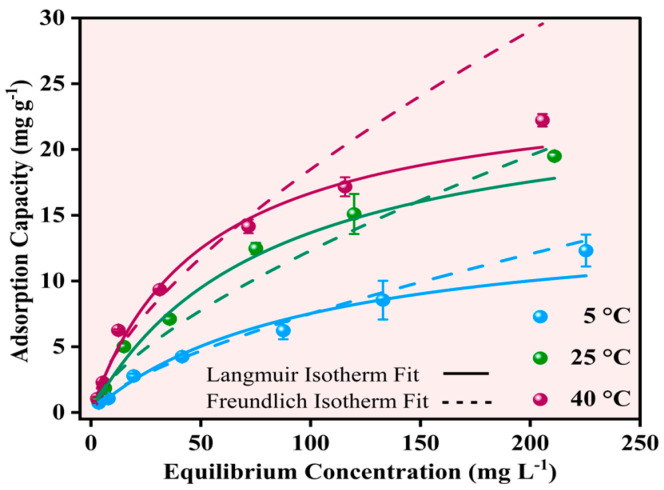
Effect of Pb^2+^ equilibrium concentrations of Pb^2+^ adsorption capacities at 5, 25, and 40 °C using Langmuir (solid line) and Freundlich (dashed lines) isotherms.

The equilibrium adsorption behavior of Pb^2+^ onto Fe-Ti mixed oxide NP was investigated at three temperatures (5, 25, and 40 °C) using initial concentrations in the range of 5–250 mg L^−1^. The experimental data were analyzed using Langmuir and Freundlich isotherm models to understand the adsorption mechanism and capacity. The Langmuir isotherm model showed superior fit across all temperatures (solid lines in Figure 10) compared to the Freundlich model (dashed lines, Figure 10), with higher R^2^ values of 0.99674, 0.98487, and 0.98654 at 5, 25, and 40 °C, respectively. The maximum adsorption capacities (qm) calculated from the Langmuir model increased with temperature: 14.93440 ± 2.87988 mg g^−1^ (5 °C), 24.60211 ± 2.85947 mg g^−1^ (25 °C), and 25.10185 ± 3.01452 mg g^−1^ (40 °C), indicating an endothermic adsorption process.

In contrast, the Freundlich model yielded lower R^2^ values (0.97507, 0.93506, and 0.93519) and higher Chi-square values at all temperatures, suggesting that Pb^2+^ adsorption follows monolayer coverage on the NP surface rather than multilayer adsorption [52]. The better fit of the Langmuir model aligns with the surface characteristics identified by XPS analysis, where specific binding sites, including surface OH groups and Fe^3+^/Fe^2+^ centers, contribute to defined adsorption sites. The Langmuir constant (KL) values also increased with temperature (0.01047 ± 0.00388, 0.02854 ± 0.00337, and 0.03196 ± 0.0047 L mg^−1^ at 5, 25, and 40 °C, respectively), indicating stronger adsorbate–adsorbent interactions at higher temperatures.

#### 2.2.4. Dose Optimization

**Figure 11:**
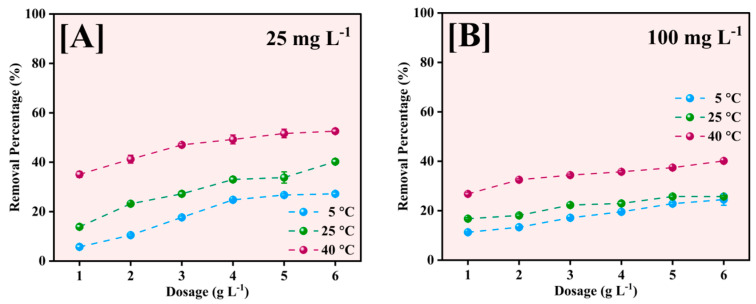
Effect of Fe-Ti mixed NP dose for Pb^2+^ removal at 5, 25, and 40 °C for 25 mg L^−1^ (**A**) and 100 mg L^−1^ (**B**) initial Pb^2+^ concentrations.

The effect of adsorbent dosage on Pb^2+^ removal efficiency was investigated at three different temperatures (5, 25, and 40 °C) for initial Pb^2+^ concentrations of 25 and 100 mg L^−1^, as illustrated in Figure 11. The adsorbent dose was varied from 1 to 6 g L^−1^ to determine optimal conditions for maximum removal efficiency.

The removal percentage increased with increasing adsorbent dose across all temperatures for the 25 mg L^−1^ initial concentration (Figure 11A). At 40 °C, the removal efficiency increased from approximately 35% at 1 g L^−1^ to 52% at 6 g L^−1^. A similar trend was observed at 25 °C, with removal efficiency increasing from 15% to 40%, while at 5 °C, the increase was more modest, from 10% to 25%. The enhanced removal at higher temperatures suggests that the adsorption process is endothermic. At the higher initial concentration of 100 mg L^−1^ (Figure 11B), the removal efficiencies were generally lower across all dosages and temperatures, indicating increased competition for binding sites at higher Pb^2+^ concentrations.

The maximum removal achieved was approximately 40% at 40 °C with 6 g L^−1^ dosage, compared to 25% and 20% at 25 °C and 5 °C, respectively. The gradual increase in removal efficiency with dosage at 100 mg L^−1^ suggests that surface saturation becomes a limiting factor at higher initial concentrations [53].

The observed temperature dependence of removal efficiency across all dosages indicates that thermal energy facilitates the adsorption process, possibly by increasing the mobility of Pb^2+^ ions and enhancing their interaction with the active sites on the NP surface. The optimal dosage appears to be 6 g L^−1^, although the incremental improvement in removal efficiency becomes less pronounced above 4 g L^−1^, particularly for the 100 mg L^−1^ concentration, suggesting that further increases in adsorbent dose may not be economically justified.

From the studies carried out here, the optimal conditions obtained with the highest adsorption capacity are listed in Table 1. This value is compared with the available literature. This comparison reveals several important trends that contextualize the advantages of our approach.

Our analysis of the literature shows that iron-based adsorbents generally demonstrate higher adsorption capacities (ranging from 14.03 to 176.33 mg g^−1^) [54,55,56,57,58,59] compared to titanium-based materials (3.65 to 7.41 mg g^−1^) [60,61]. This disparity highlights iron’s superior affinity for lead ions. However, it is worth noting that high doses of iron can present toxicity concerns, with serum iron levels above 500 mcg/dL associated with severe systemic toxicity, while titanium exhibits considerably lower toxicity profiles with current exposure levels not considered a health risk [62].

Interestingly, we identified only one previous study on Fe-Ti mixed oxide NP’s [20], which reported a considerably lower adsorption capacity (3.0 mg g^−1^) than our green-synthesized material (25.10 mg g^−1^). This significant improvement demonstrates the effectiveness of our synthesis approach. While some biochar-supported NP systems show higher capacities [10], these involve more complex synthesis procedures and additional materials.

Our Fe-Ti mixed oxide NP strike an optimal balance between performance, synthesis simplicity, and reduced toxicity concerns. By combining titanium’s structural stability and lower toxicity with iron’s strong affinity for lead [63], we have developed an environmentally responsible adsorbent with competitive performance. The green synthesis approach using dextrose further enhances the sustainability profile of our material, addressing a critical gap in environmentally benign water treatment technologies.

## 3. Materials and Methods

### 3.1. Chemicals

All chemicals were used as received without further purification. Titanium(IV) n-butoxide (Ti(OBu)_4_, ≥99%, 340.36 g mol^−1^) was obtained from Thermo Scientific (Waltham, MA, USA). Iron(III) chloride (FeCl_3_, ≥99%, 162.2 g mol^−1^), and lead(II) nitrate (Pb(NO_3_)_2_, ≥99.0%, 331.2 g mol^−1^) were purchased from Sigma-Aldrich (St. Louis, MO, USA). Dextrose anhydrous (C_6_H_12_O_6_, ≥99.5%, 180.16 g mol^−1^) was procured from Merck KGaA (Darmstadt, Germany). Ultra-pure water was used throughout all experiments.

### 3.2. Synthesis of Fe-Ti Mixed NP

The Fe-Ti mixed NP composite was synthesized using a modified green synthesis method [64]. Initially, 20.00 mL of 2.93 mol L^−1^ Ti(OBu)_4_ was mixed with ~20 mL of ethanol in a beaker and stirred at 20 rpm for 2 min. Following this, 40.00 mL of 0.1 mol L^−1^ FeCl_3_ solution in ethanol was added and stirred for 5 min. Subsequently, 0.4% (*w*/*v*) dextrose solution was added dropwise (5 mL min^−1^) while stirring until a yellow gel was formed. The resulting gel was dried overnight at 60 °C to obtain the final NPs [20,65].

### 3.3. Characterization of Fe-Ti Mixed NP

#### 3.3.1. SEM and EDS

SEM and EDS analyses were performed using a scanning electron microscope JEOL LSM-6010LA (Tokyo, Japan) and In TouchScope software (version 1.11) [66]. The samples were prepared on carbon-coated adhesive tape. Backscattered electron images were collected using an accelerating voltage of 10 kV and a load current of approximately 90 µA with a working distance of 9 mm. EDS spectra were gathered at a magnification of 2000×, and the analyzed area was 0.15 mm^2^ (110 µm × 135 µm). A silicon-drift detector performed qualitative analysis and quantification of the elemental composition with the characteristic X-rays. Semi-quantification was based on theoretical correction of the ZAF (Z-atomic number, A-absorption, and F-fluorescent excitation) effect.

#### 3.3.2. FTIR

The FTIR spectra of the synthesized Fe-Ti mixed oxide NPs were obtained in the 400–4000 cm^−1^ wavenumber range using a thermo IS5 FTIR with ID7 Diamond ATR Spectrometer (Thermo Scientific, Waltham, MA, USA) with attenuated total reflection (ATR). The Fe-Ti mixed oxide NP sample was applied to the diamond crystal without posttreatment. The spectral peaks were analyzed using Omnic FTIR 9.13 software (Thermo Scientific, Waltham, MA, USA).

#### 3.3.3. XRD and TGA

XRD analysis was performed using a Bruker Endeavor X-ray diffraction (Madison, WI, USA) with a 1 kW Cu X-ray tube operated at 40 kV and 25 mA. The wavelength was 1.540598 Å, and measurements were collected against coupled θ/2θ scan type. The diffraction patterns were collected to analyze the NPs’ crystalline structure and phase composition. TGA was performed using a Perkin Elmer Diamond TG/DTA instrument (Shelton, CT, USA). Approximately 5 mg of the synthesized Fe-Ti mixed oxide nanoparticle sample was placed in a platinum crucible. The temperature program was set from 30 °C to 1000 °C with a heating rate of 10 °C min^−1^. The weight loss percentage was recorded as a temperature function to determine the material’s thermal stability and decomposition behavior.

#### 3.3.4. XPS

XPS measurements were conducted using a OmicronESCA+ spectrometer (Scienta Omicron, Taunusstein, Germany) with a Mg X-ray source operating at 300 W. The operating pressure was maintained at 2 × 10^−9^ torr. Low-energy electrons were used for charge compensation. All spectra were charge-referenced by adjusting the C1s peak to 284.6 eV. Survey scans were acquired at a pass energy of 120 eV, while high-resolution spectra were collected at 20 eV pass energy. Data deconvolution and analysis were performed using Origin Pro version 10.2.

#### 3.3.5. Surface Area Analysis

The BET surface area was determined using Anton paar Nova 600 (Gasse 1, 8042 Graz, Austria) with nitrogen adsorption–desorption measurements. Samples were degassed for 6 h at 130 °C before analysis to ensure the surface was free of gas molecules for surface area analysis.

### 3.4. Adsorption Studies

All experiments were performed in batch mode in a 50 mL polypropylene tube. A 50.0 mg quantity of Fe-Ti mixed oxide NPs was equilibrated with 25.00 mL of lead ion (Pb^2+^) solutions at 200 rpm and 25 °C except specified conditions mentioned below. All the experiments were replicated three times, and the error was represented as the standard deviation.

#### 3.4.1. Determination of Point of Zero Charge and Effect of Solution pH

The pH drift method determined the PZC of Fe-Ti mixed oxide NPs. The solution pHs were measured using a Mettler Toledo pH meter, and the solution pH changes were plotted against the initial pH values of 0, 1, 3, 5, 7, 9, 11, and 13 in 0.01 mol L^−1^ NaNO_3_ matrix after equilibrating with the NPs.

#### 3.4.2. Kinetic Studies

Adsorption kinetics were studied using 25.00 mL solutions at pH 5 containing 5, 25, and 100 mg L^−1^ Pb^2+^ with 50.0 mg of NPs. Samples were filtered after 2, 5, 10, 15, 30, 60, 120, and 180 min equilibration intervals.

#### 3.4.3. Isotherm Studies

Isotherm experiments were conducted with a contact time of 180 min at three different temperatures (5 °C, 25 °C, and 40 °C) at pH 5 for initial 5, 10, 25, 50, 100, 150, and 250 mg L^−1^ Pb^2+^ concentrations.

#### 3.4.4. Dose Optimization

The effect of adsorbent dosage was investigated using 25.00 mL of 25 and 100 mg L^−1^ Pb^2+^ solutions at 5 °C, 25 °C, and 40 °C. Adsorbent doses of 5, 25, 50, 100, 150, and 250 mg were tested.

#### 3.4.5. Analytical Methods

The remaining Pb^2+^ concentrations were determined using Varian 220 FS Atomic Absorption Spectroscopy (AAS) (Mulgrave, Victoria, Australia). Samples were filtered using Whatman filter papers through gravitational filtration. 5, 10, 15, 20, and 25 mg L^−1^ Pb^2+^ calibration standards were prepared in 2% HNO_3_. Samples were appropriately diluted with 2% HNO_3_ before analysis. The adsorption capacity at equilibrium (Q_e_, mg g^−1^) was calculated using the following equation (Equation (1)):

(1) $$Q_{e}=\frac{\left(C_{0}-C_{e}\right)\times V}{m}$$

where C_0_ is the initial concentration of Pb^2+^ (mg L^−1^), Cₑ is the equilibrium concentration of Pb^2+^ (mg L^−1^), V is the volume of the solution (mL), and m is the mass of the adsorbent (g).

## 4. Conclusions

This study demonstrates the successful green synthesis and application of Fe-Ti mixed oxide NPs for efficient Pb removal from aqueous solutions. The synthesized NPs exhibited an amorphous structure with a high surface area (292.89 m^2^ g^−1^) and mesoporous characteristics, confirmed through comprehensive characterization using XRD, BET, and microscopy techniques. XPS analysis revealed complex surface chemistry with mixed Fe^3+^/Fe^2+^ valence states in a Ti^4+^-rich framework, providing diverse binding sites for Pb adsorption. The NPs’ surface properties, including abundant OH groups and a low point of zero charge (0.22), created favorable conditions for Pb binding through multiple mechanisms, including electrostatic interactions and surface complexation.

The material demonstrated exceptional adsorption performance, achieving optimal removal at pH 5 with a maximum adsorption capacity of 25.10 mg g^−1^ at 40 °C. Kinetic studies revealed rapid initial uptake following the pseudo-second-order model (R^2^ > 0.99), indicating chemisorption as the dominant mechanism. The Langmuir isotherm model provided the best fit (R^2^ > 0.98), suggesting monolayer adsorption on homogeneous surface sites. The endothermic nature of adsorption was evidenced by enhanced removal efficiencies at elevated temperatures, while dose optimization studies revealed optimal performance at 6 g L^−1^ adsorbent concentration.

These findings establish Fe-Ti mixed oxide NPs as a promising adsorbent for Pb removal, with their green synthesis route offering an environmentally sustainable approach to water treatment applications. The synergistic combination of Fe’s high affinity for heavy metals and Ti’s structural stability, coupled with the advantageous surface properties induced by the green synthesis method, resulted in a highly effective adsorbent material. Future studies should focus on investigating the material’s performance in complex water matrices and exploring potential scale-up strategies for practical applications in water treatment systems.

## Figures and Tables

**Table 1:** Pb(II) adsorption capacities along with other experimental conditions (pH, contact time, and equilibrium concentration range) versus Fe oxides, Ti oxides, and mixed Fe and Ti oxides.

Adsorbent	Maximum Adsorption Capacity (mg g^−1^)	pH	Temperature (°C)	Reference
Fe-Ti mixed oxide nanoparticles (current study)	25.10	5.0	40	Present work
Goethite (FeOOH)	15.11	6.0	30	[54]
O-Fe_3_O_4_ nanoparticles	30.87	5.5	15	[55]
HBC (biochar with nanoparticles)	146.84	6.0	30	[10]
Fe_3_O_4_—(PoPs) coated NPs	51.81	5.7	25	[56]
α-Fe_2_O_3_ nanoparticles	24.00	5.5	25	[57]
Sulfur-modified iron nanoparticles	14.03	3.0	25	[58]
MNPs-Iron	176.33	5.0	35	[59]
Nano TiO_2_	7.41	6.0	25	[60]
W-doped TiO_2_	3.65	5	25	[61]
Composite Fe-Ti oxides nanoparticles	3.0	7	25	[20]

## Data Availability

Data are available within the article and will be made available upon request.

## Supplementary Materials

The following supporting information can be downloaded at: https://www.mdpi.com/article/10.3390/molecules30091902/s1, Table S1: XPS data.

## Abbreviations

The following abbreviations are used in this manuscript:

AAS	Atomic Absorption Spectroscopy
BET	Brunauer–Emmett–Teller
Cu	Copper
EDS	Energy Dispersive X-ray Spectroscopy
Fe	Iron
FeCl_3_	Iron(iii) chloride
FTIR	Fourier transform infrared spectroscopy
NPs	Nanoparticles
O	Oxygen
OH	Hydroxyl
Pb	Lead
Pb(NO_3_)_2_	Lead(ii) nitrate
ppb	Parts per billion
PZC	Point of zero charge
SEM	Scanning electron microscopy
Ti	Titanium
Ti(OBu)_4_	Titanium(IV) n-butoxide
TGA	Thermogravimetric analysis
TiO_2_	Titanium dioxide
XPS	X-ray photoelectron spectroscopy
XRD	X-ray diffraction
