# Supplementary material for: Green Synthesis and Characterization of Fe-Ti Mixed Nanoparticles for Enhanced Lead Removal from Aqueous Solutions
Source: Molecules. 2025 Apr 24;30(9):1902. doi: 10.3390/molecules30091902 (PMC12074276; doi:10.3390/molecules30091902)
Supplement: Supplementary file 1 [file molecules-30-01902-s001.zip › molecules-3576208-supplementary.pdf]

Supplementary Information

Green Synthesis and Characterization of Fe-Ti Mixed Nanoparticles for Enhanced Lead Removal from Aqueous Solutions

Shamika P. W. R. Hewage and Harshica Fernando *

Department of Chemistry Prairie View A&M University, Prairie View, TX 77446, USA
* Correspondence: hufernando@pvamu.edu; Tel: (936) 261-3112; Fax: (936) 261-3117

Table S1- XPS data

Element	Peak No.	Binding Energy (eV)	Binding Energy range (eV)	Atomic %	Assigned functionality
Fe2p	I	710.13	709-711	0.49%	Fe^{3+} in Fe_2O_3
	II	714.23	713-715	0.05%	Fe^{2+}/Fe^{3+} satellite
	III	718.61	718-719	0.19%	Fe^{3+} satellite
	IV	723.37	722-724	0.13%	Fe^{3+} in Fe_2O_3
	V	726.10	725-727	0.11%	Fe^{2+}/Fe^{3+} satellite
Ti2p	I	458.60	458-459	13.04%	Ti^{4+} in TiO_2 ($2p_3/_2$)
	II	464.29	463-465	6.26%	Ti^{4+} in TiO_2 ($2p_1/_2$)
O1s	I	529.88	529-530	25.22%	Metal oxide (M-O)
	II	530.99	530-532	20.45%	Surface hydroxyl/OH groups
C1s	I	284.10	283-285	11.36%	C-C, C-H bonds
	II	285.45	285-286	10.93%	C-O bonds
	III	288.21	287-289	2.92%	O-C=O (carboxyl)

Element	B.E. (eV)	Intensity (CPS)
C1s	288.21	5,547.53
C1s	285.45	5,461.43
C1s	284.10	9,730.63
Fe2p	726.10	22,818.13
Fe2p	723.37	23,318.75
Fe2p	718.61	22,871.50
Fe2p	714.23	23,908.75
Fe2p	710.13	22,563.75
O1s	530.99	49,751.50
O1s	529.88	50,027.00
Ti2p	464.29	22,794.20
Ti2p	458.60	38,386.30
